# Intrasphenoidal encephalocele: an incidental finding in emergency department

**DOI:** 10.1186/1865-1380-6-45

**Published:** 2013-12-05

**Authors:** Muhammad Waseem, Ruchi Upadhyay, Husayn Al-Husayni, Samuel Agyare

**Affiliations:** 1 Lincoln Medical & Mental Health Center, 234 East 149th Street, Bronx, NY 10451, USA

**Keywords:** Encephalocele, Intrasphenoidal encephalocele, Basal encephalocele

## Abstract

**Background:**

Basal encephaloceles are rare clinical entities. Intrasphenoidal encephalocele (ISE) is a rare form of basal encephalocele. The clinical presentation is often subtle and may remain undetected. Only a limited number of cases of ISE have been reported, mostly in middle-aged patients who presented with symptoms. Clinical diagnosis and management remain a challenge. Our case reports ISE as an incidental finding in a very elderly male patient.

**Findings:**

We present a case of ISE discovered as an incidental finding in a 99-year-old male who presented to the emergency department (ED) after a fall from bed. The encephalocele was discovered in the evaluation of the computed tomography (CT) scan of the head.

**Conclusion:**

This is an incidental manifestation of a rare disease. It demonstrates that these encephaloceles may not manifest clinically or symptomatically as other reports have suggested.

## Findings

### Introduction

Basal encephaloceles are rare clinical entities. The clinical presentation is often subtle and may remain undetected. We present a case of intrasphenoidal encephalocele discovered as an incidental finding in a 99-year-old male who presented to the emergency department (ED) after a fall from bed. The encephalocele was discovered in the evaluation of computed tomography (CT) scan of the head. This is an incidental manifestation of a rare disease. It demonstrates that these encephaloceles may not manifest clinically or symptomatically as other reports have suggested.

### Description

A 99-year-old male presented to the ED after he fell from his bed. History was obtained from the home attendant, who accompanied the patient, as the patient was non-communicative because of severe dementia. As per the home attendant, the patient fell from his bed from a height of approximately 4 feet. The attendant noticed a swelling on the forehead of the patient. The patient remained dazed for a while but there was no vomiting or abnormal movement. His past medical history was significant for Alzheimer’s dementia, hypertension, diabetes mellitus, and benign prostatic hyperplasia.

In the ED, he was in no apparent distress and was at his usual baseline mental status. His vital signs were as follows; temperature 36.5°C (97.8°F), heart rate 82 beats per minute, respiratory rate 20 breaths per minute and blood pressure 125/60 mmHg. On physical examination, a soft tissue hematoma was noted on the forehead. His pupils were equal and reactive, and extraocular movements were intact. A linear 2-cm laceration was noted on his right periorbital region. There was no hemotympanum. There was no neck tenderness. His chest was clear with bilateral breath sounds. His abdomen was soft and non-tender. There was no swelling or tenderness over his extremities. The remainder of the physical examination was unremarkable.

**Figure 1:**
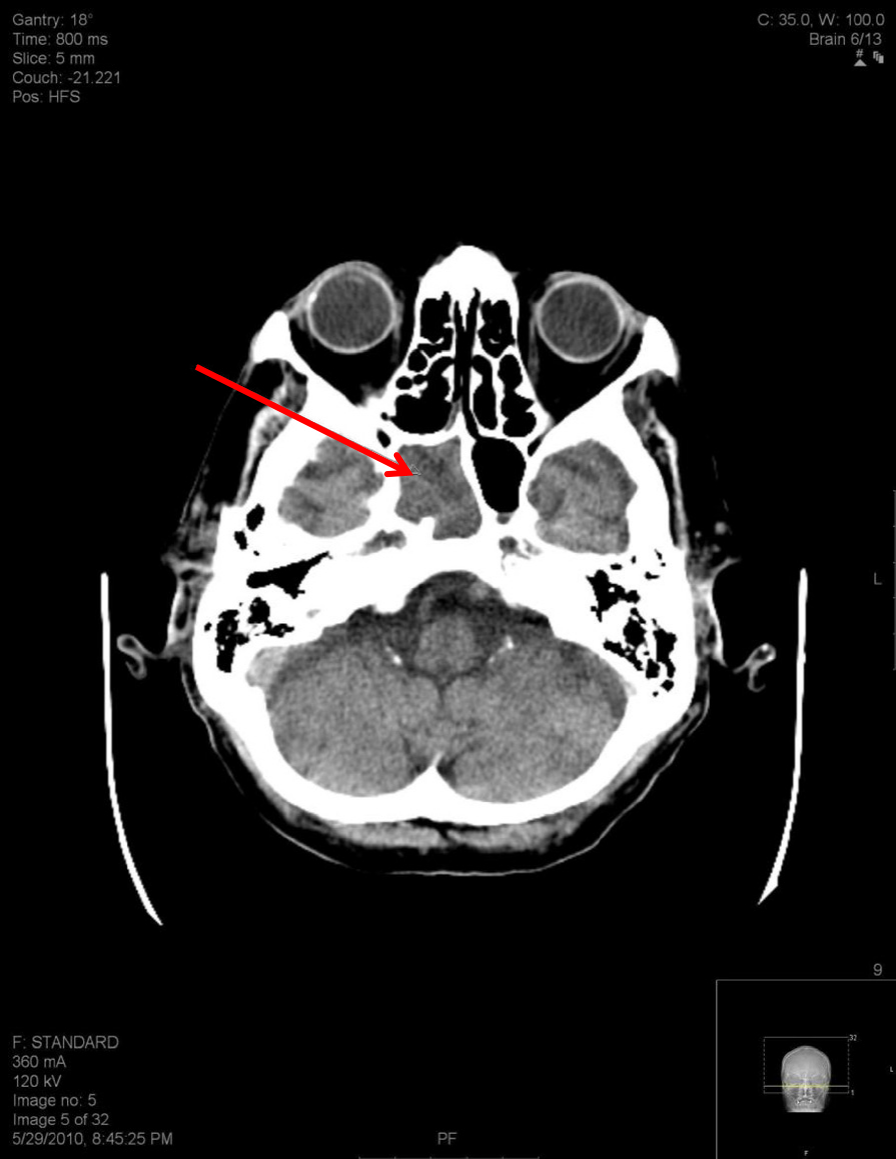
Axial view of brain material within the right side of the sphenoid sinus.

**Figure 2:**
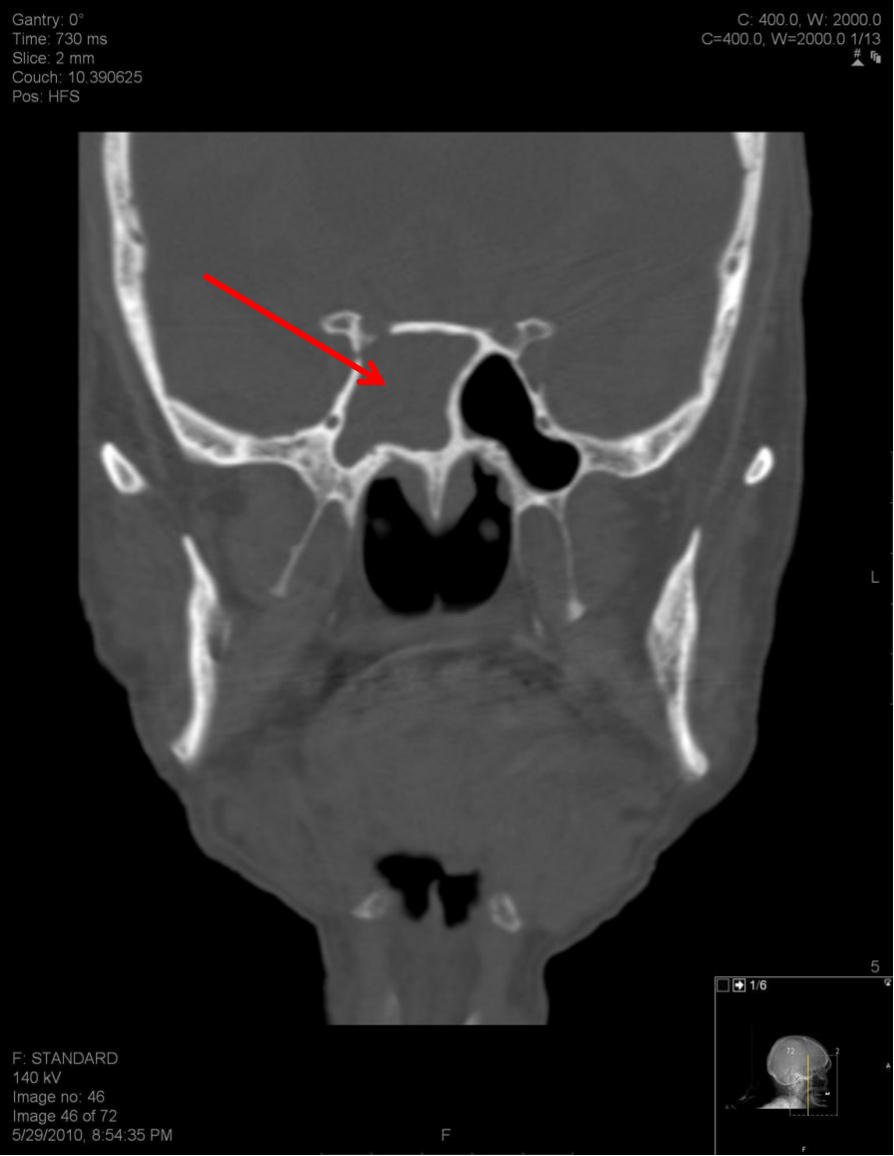
Coronal view of brain material within the right side of the sphenoid sinus.

A CT scan of the head and facial bones was obtained. The images demonstrated severe dilation of the ventricles and sulci suggestive of cerebral and cerebellar cortical atrophy. Also noted was a new right parietal white matter hypodense (ischemic) lesion and brain material within the right side of the sphenoid sinus suggestive of an intrasphenoidal encephalocele (Figures 1 and 2).

## Discussion

Intrasphenoidal encephalocele [ISE] is a rare form of basal encephalocele. Only a limited number of cases of ISE have been reported; among these, most were reported in middle-aged patients who presented with findings of cerebrospinal fluid rhinorrhea [1] and more often in females than males [2]. Our case reports intrasphenoidal encephalocele as an incidental finding in an elderly male patient.

Encephaloceles are divided into two types: anterior and posterior. Posterior encephaloceles account for three-fourths of those described and anterior for only about 1.5% of all encephaloceles [3]. On the basis of the location of the bone defect, basal encephaloceles are further subdivided into transphenoidal, transethmoidal, spheno-ethmoidal and spheno-orbital [4].

Though intrasphenoidal encephaloceles are classified as a type of transphenoidal encephalocele, they should be considered a completely separate entity [5]. Intrasphenoidal encephaloceles (extending into but not through the sphenoid sinus) and true transphenoidal encephaloceles have different morphological and clinical features as well as different etiologies [3,6]. The etiology of the ISE can be attributed to “trauma, tumor, intracranial infection, or a surgical procedure involving the sellar region, the parasellar region, or the paranasal sinus” or a small defect in the middle cranial fossa [7]. They also differ in their surgical treatment [5]. ISE is further subclassified, based on its location, into the perisellar and lateral-recess type [4].

The clinical presentations of ISE are caused by the involvement of the structures surrounding the sinus [8]. The most common presentation is CSF rhinorrhea, but the clinical presentation can often be complicated by recurrent meningitis, chronic headache, orbital pain, cranial neuropathy and subdural hematomas [6]. A good history and physical examination cannot be substituted for any other diagnostic modalities [8]. Myssiorek and Cohen [8] also emphasized that the diagnostic workup of ISE should comprise sinus radiographic studies, computed tomography scan with contrast and finally excisional biopsy to confirm the diagnosis. CT scans are limited in that they cannot differentiate among herniating brain, mucosal thickening, a retention cyst or a potential aneurysm, and MRI is more reliable for diagnosing and management [4].

Surgical management of basal encephaloceles should be preceded by careful evaluation and establishing the location of the defect [6]. Extracranial and intracranial approaches have been advocated for surgical management, and both have their own limitations [9]. Irrespective of the approach, the goals of the treatment of basal encephalocele are removal of the herniated sac, preservation of functional neural tissue, closure and reconstruction of the defect with healthy tissue, and restoration of the CSF pathway with shunting [6]. The presence of persistence rhinorrhea, epipharyngeal respiratory obstruction or progression of neurological deficits necessitates the use of a surgical approach [10].

Since intrasphenoidal encephaloceles are such rare clinical entities, their diagnosis and management remain a challenge. Our case report demonstrates a completely asymptomatic presentation, which further emphasizes the necessity for radiological diagnosis.

## Abbreviations

ED: Emergency department; ISE: Intrasphenoidal encephalocele.

## Competing interests

The authors declared that they have no competing interests.

## Authors’ contributions

MW contributed to the concept, design and revision for important intellectual content. RU carried out the acquisition of data and drafting of the manuscript. HA contributed to the radiological findings and related intellectual content. SA gave final approval of the version to be published. All authors read and approved the final manuscript.
